# Maternal Shared Pleasure, Infant Withdrawal, and Developmental Outcomes in a High Risk Setting in South Africa

**DOI:** 10.3389/fpsyt.2021.668009

**Published:** 2021-07-20

**Authors:** Anusha Lachman, Marlette Burger, Esmè R. Jordaan, Jukka Leppanen, Kaija Puura, Dana J. H. Niehaus

**Affiliations:** ^1^Department of Psychiatry, Stellenbosch University, Stellenbosch, South Africa; ^2^Physiotherapy Division, Department of Health and Rehabilitation Sciences, Stellenbosch University, Cape Town, South Africa; ^3^Biostatistics Unit, South African Medical Research Council, Durban, South Africa; ^4^Statistics and Population Studies, University of the Western Cape, Cape Town, South Africa; ^5^Department of Psychology and Speech-Language Pathology, University of Turku, Turku, Finland; ^6^Department of Child Psychiatry, Tampere University, Tampere, Finland

**Keywords:** shared pleasure, infant withdrawal, positive interactions, mother infant interaction, infant development, synchrony

## Abstract

**Background:** Infants in lower middle income countries are often exposed to early adversities which may lead to suboptimal caregiving environments and place them at risk of not achieving their developmental potential. Synchrony and positive engagement in the mother-infant relationship plays a critical role in buffering the impact of early adversity. Shared Pleasure (SP) is considered a marker of high intensity positive interaction and may hold a promise of improving developmental outcomes.

**Methods:** This study was part of a prospective observational study of mothers with and without mental illness in South Africa. Dyadic videos were assessed for SP and infant withdrawal (using the Alarm Distress Baby Scale) at 6 months. Infant developmental outcomes were assessed using the Bayley's Scales for Infant and Toddler Development, third edition at 18 months.

**Results:** Ninety-one dyads were assessed for SP. The occurrence of SP was low (20%). There was no significant association with an EPDS measure of maternal depression (*p* = 0.571) and SP moments. Infant withdrawal was high (72%) and associated with male infant gender (*p* = 0.025). There was a significant association between the occurrence of SP and a lower score of infant withdrawal (estimate = −1.29; SE = 0.4; *p* = 0.0002). The number of SP moments at 6 months was significantly associated with motor (estimate = 2.4; SE = 0.9; *p* = 0.007) and marginally significant with cognitive scores (estimate = 1.9; SE = 1.0; *p* = 0.052) at 18 months. Regression modelling differential outcomes showed a greater improvement in cognitive scores at 18 months in infants with an SP moment compared to those without an SP moment [SP average difference (AD) = 7.4 (2.4), no SP AD = 10.4 (1.2); *p* = 0.012]. Infants without an SP moment experienced a larger decrease in motor scores at 18 months compared to those with an SP moment [SP AD = −3 (3.0); no SP AD = −10.6 (1.5), *p* = 0.027].

**Conclusion:** While the occurrence of SP in this sample was low and the rates of infant withdrawal were high, there were promising results suggesting early positive SP interactions may contribute to improvements in subsequent developmental outcomes.

## Background

The 2016 Lancet Global Health report estimated that in lower middle-income countries (LMICs), up to 250 million children under the age of 5 risk falling short of their full developmental potential due to early exposure to adversities (1). Sub-Saharan Africa recorded the highest prevalence (66%) of children at risk of not reaching their developmental potential (1). The effects of maternal mental health and poverty are especially influential early in life when positive experiences are more likely to contribute to the development of synaptic connexions important for development, and negative experiences can shift a child off the optimal developmental trajectory.

Environmental adversity, particularly caregiver mental illness and toxic stress, may contribute to the disruption of normal developmental processes (2, 3). Infants born in such circumstances are considered as high risk and susceptible to psychosocial and physical developmental challenges (4). Multiple interacting domains such as health, nutrition, responsive caregiving, and early stimulation are needed to promote not only physical growth but also to enhance social and emotional development in infancy (3, 5). Responsive caregiving in this context refers to the ability of the caregiver to make eye contact with, to model and encourage interaction while responding appropriately to the infant, and is considered a foundational component of the nurturing care that infants require in order to thrive.

### Environmental Risk and Depression/Outcomes

Infants from poorer communities are especially vulnerable to maternal mental illnesses as they are often subject to sub optimal caregiving environments that are strained under social adversity, substance use, and high rates of depression (6, 7). Tomlinson et al. (8) reported maternal depression rates as high as 35% in Khayelitsha, a lower socio-economic community in Cape Town, South Africa. In this setting, maternal mental illness and substance abuse have been linked to lower infant engagement with their mothers (6) and by inference, a poorer experience of responsive caregiving (9). Infants of depressed mothers have also demonstrated significantly lower cognitive and social emotional competence and compromised growth parameters (10–12). It is important however to note that depression in the mother does not necessarily mean that the infant interaction will definitely be impaired, as this interaction also depends on individual infant characteristics and can often be buffered by environmental support from a secondary or alternate caregiver (13). The parent-infant relationship itself plays a critical role in buffering the impact of early adversity on long term stress responses and developmental trajectories (14). Evidence relating to the regulation of stress hormones in young children suggests that the presence of a supportive adult caregiver strongly regulates and in effect buffers the stress response (15, 16).

### Parent/Infant Interaction

From as early as 2 months of age, infants demonstrate skills that support engagement and interaction with their caregivers as part of a social interaction (17, 18). Amongst these biologically determined skills, the ability to initiate and maintain eye contact and the use of facial expressions to engage the caregiver, serves as part of early social communication. Synchrony, which is described as the face to face mechanism mothers use for building and maintaining positive affect with their infants within the early mother-infant relationship, supports the optimal development of infants (19, 20). In particular, face-to-face interactions may elicit a positive effect on both the infant and the mother (21). During episodes of mutual gaze, the mother and infant may engage in spontaneous facial, vocal, and gestural communications. Such highly arousing, face-to-face interactions allows the infant to be exposed to high levels of social and cognitive information (19, 22). Direct gaze strengthens bi-directional adult-infant neural connectivity during this communication (23) and is associated with higher autonomic arousal that may indicate a stronger intensity of the shared interactional experience (24). Maternal behavioural interactions are also associated with the amount of expressed positive emotions (19, 25).

Field et al. (26) reported that depressed mother-infant dyads expressed less positive emotions and spent more time in negative behavioural states. A meta-analysis by Goodman et al. (27) also found an association with negative child affectivity and maternal depression. Puura et al. (28, 29) hypothesised that the simultaneous sharing of a smile with direct gaze contact between mother and infant was a marker of high intensity positive affectivity and named this paradigm Shared Pleasure (SP). SP has since been shown to correlate with attachment security (28) and is considered highly malleable in the first 12 months of life (30), which holds promise for dyads where the positive interaction is less than optimal. SP has also been applied in a culturally diverse sample in South Africa where SP was experienced more frequently in married mothers (*p* = 0.016) and significantly more Black African mothers (*p* = 0.033) (31). That study also found a significant relationship between the presence of SP moments and the absence of mental illness in the mothers (*p* = 0.021).

### Infant Withdrawal

Social behaviour during infancy refers to the degree and style of responsiveness to social stimuli (32). Infant withdrawal is part of the infant's normal regulatory capacity (33) that serves to transiently regulate the flow of stimulation when the infant is tired or over stimulated after a positive encounter (34). It is normal for infants to display brief social withdrawal behaviour like closing their eyes or turning away, which helps them to regulate (33, 35). Even very short episodes of non-responsiveness on the part of the caregiver, may initiate withdrawal or protest reactions from a normally developing infant (36). Social withdrawal, indicated by a lack of either positive or negative behaviours, is an alarm signal of infant distress regardless of the cause (37) and may alert the clinician to the possibility that the infant is not displaying age-appropriate emotional or social behaviour (32). Sustained or persistent withdrawal behaviours are not normal and infants may make less eye contact, smile or vocalise less during interactions which may be an early warning of lack of synchronicity with their parents (37, 38). Increased social withdrawal behaviour is associated with both biological risk factors (such as preterm deliveries) and environmental stressors including maternal psychiatric disorders (such as depression and anxiety) (19, 37). Studies of the relationship between infant withdrawal behaviours and maternal depression have been mixed. Mäntymaa et al. (39) reported infant withdrawal in a low risk sample where the parents' perceived mental health was associated with infant social withdrawal, while other studies did not find an association with current depressed maternal mood and withdrawn infant behaviours (32, 35). There are however higher distributions of infant withdrawal in higher risk samples of infants with sleeping, eating or crying difficulties, or problems within the parent-infant relationship (38, 40). Sustained social withdrawal may also be associated with serious developmental, physical and emotional disorders in infants (41, 42). In an Australian study by Milne et al. (43), significant negative correlations were found between infant social withdrawal and Cognitive and Language scales on the Bayley Scales of Infant and Toddler Development. Higher scores of social withdrawal behaviour were also found to be associated with delays in reaching language and motor milestones in infants in a longitudinal French birth cohort (44). These studies provide some support for the association of infant withdrawal and later infant development.

The relationship between positive maternal infant engagement and its impact on infant withdrawal and developmental outcomes has not received much attention in LMIC settings. In environments of high psychosocial adversity, there is room for the exploration of and promotion of positive maternal infant interactions if they hold potential to influence better infant outcomes.

The current study aimed to assess the association between early SP moments, withdrawn infant behaviour, and its relationship to later infant neurodevelopmental outcomes in a high risk sample of South African mothers and their infants.

## Materials and Methods

### Study Design

This study formed part of a larger prospective observational study, The Maternal and Infant Mental Health (MIMH) study, based at Stikland Psychiatric Hospital (Bellville, Western Cape, South Africa). The MIMH study aims to investigate the impact of maternal mental illness and infant measures of social cognitive deficits and oto-acoustic characteristics on mother–child interactions and attachment. The parent MIMH study recruited mothers with and without current mental illnesses attending a state and private clinic. Potential participants were recruited for participation from the maternal mental health clinic at the hospital and were screened first by the research team for capacity to provide informed consent before participation. The current study used the data collected from the state clinic between 2014 and 2019. Depending on the quality of the videos, and the presence of infant developmental data, 91 mother infant pairs out of a possible 117 were suitable for assessment of infant and maternal interactions. Babies were videotaped and assessed at their 6-month visit. At the time of the video recordings of SP moments, the dyads had not received any therapeutic intervention together. Mothers with mental illnesses were known to mental health services and received mental health care, but babies were only seen prior to participation in the developmental assessments. Any babies identified as being at risk or of concern, were referred to the psychologist or the local clinic for further intervention.

### The Inclusion of Race

The referral to Racially classifying terms in this study has been used as defined in the Employment Equity Act No.55 of 1998 (45). These terms were used to provide context to the background of the historically marginalised participants and is not meant to infer any sociocultural constructs in general about these population groups.

### Tools

#### Shared Pleasure (SP)

SP was defined as the parent and the child sharing positive affects in synchrony (46). This had to be expressed in a facial expression as the curving of the mouth into a smile or laugh with gaze contact and a simultaneous or synchronised beginning and ending. SP moments were assessed from the first 5 min of a single recorded segment of the dyadic free-play video recordings of mothers and infants. Mothers were asked to play with their infants in an unstructured, undirected unrestricted free play situation as part of the Maternal and infant mental health study. Mothers were encouraged to play spontaneously with no restrictions or directions pre-empted. The first 5 min are considered sufficient for the assessment of positive interactional styles where interrater reliability is good (47). SP moments between mother and baby were measured by a single rater (AL) with the rater blinded to the psychiatric history of the mother. The measurement for SP consisted of three components: the occurrence of an SP moment, the total number of SP moments, and the duration of an SP moment. To assess interrater reliability for this study, 10% of the videos were randomly selected using the random number generator in Excel and were rated independently by two coders (AL and KP). The kappa values for the SP variables used in the analysis, i.e., the occurrence of SP sequences and the mean duration of SP (<0.5 or >0.5 s), were 1.00 and 0.78, respectively. The respective rates of interrater agreement were 100 and 87.5% which may be regarded as strong (48). None of the SP variables showed a statistically significant difference between the raters, thus showing excellent reliability.

#### Infant Withdrawal Behaviour

Infant social withdrawal was assessed using the Alarm Distress Baby Scale (ADBB) (41). This clinical instrument is aimed at evaluating social behaviours that can be easily observed during a brief observation of children 2–24 months of age. The ADBB Scale that measures sustained infant withdrawal has been used in several high income countries (20, 44), in addition to the local South African context (49, 50). The behaviours are organised into eight items: (1) Facial Expression, (2) Eye Contact, (3) General Level of Activity, (4) Self-Stimulating Gestures, (5) Vocalisations, (6) Response to stimulation, (7) Relationship, and (8) Attraction. Each item is rated on a scale from 0 (*no unusual behaviour*) to 4 (*severe unusual behaviour*). Guedeney and Fermanian (41) used a cut-off threshold score of 5 and reported a sensitivity of 0.82, a specificity of 0.78, and construct validity measures varying from 0.63 to 0.67. Similar results have been reported from several studies using the ADBB in different cultures, including South African cohorts (50, 51). A threshold cut-off of ≥5 was used as it has shown optimal sensitivity and specificity to detect infants at risk. The PI was trained by the last author (KP) and achieved reliability in the ADBB in Finland. Videotaping each assessment allowed for review of the ADBB scoring by two local accredited scorers (AL and a trained research assistant) who reviewed 10% of the more difficult assessments to achieve consensus.

#### Infant Development

Infant development was assessed using the Bayley Scales of Infant and Toddler Development, Third Edition (BSID-III) (52), a gold-standard observational measure of development for children from 0 to 42 months. It has been validated in a South African population and found to be culturally appropriate without modifications (53), although the tool has been found to slightly underestimate the developmental delay in this population (54). The tool measures development by direct observation across five subscales: cognition, language (receptive and expressive), and motor (fine and gross). These scales were measured by direct observation by a trained physiotherapist (MB) blinded to the child and family risk factors. The socio-emotional and adaptive behaviour subscales were assessed by direct observation as well as caregiver report. Quality control and monitoring processes were implemented to ensure accuracy. Age-standardised composite cognitive, motor and language scores were generated from cognitive, fine and gross motor, language, socio-emotional, and adaptive behaviour subscale scores using BSID-III normative and conversion tables, which account for gestation at delivery.

#### Maternal Mental Health

The Mini International Neuropsychiatric Interview (MINI version 5.0.0) (55), a brief, structured diagnostic interview for DSM-IV and ICD-10 psychiatric disorders was administered to parents as part of the MIMH study. The Edinburgh Postnatal Depression Scale (EPDS) (56) and the Recent Life Events Questionnaire (RLEQ) (57) were recorded individually as part of the parent study.

The EPDS is a 10-item self-report measure of recent depressive symptoms. Each item is scored on a frequency scale ranging from 0 to 3. A total score is then obtained by summing individual item responses with a higher score indicative of more severe depressive symptoms. A cut-off score of ≥12 has been used to indicate probable depression as has previously been validated in a South African cohort of women (58).

The RLEQ was derived from the List of Threatening Experiences (LTE) with the aim of devising a more practical and cost-effective tool for use in psychological, social, and psychiatric populations (57). It is a 21-item self-report scale, assessing whether a person or someone close to him/her experienced specific stressful life events (such as serious illness, injury, death, unemployment, etc.) during the past 12 months. The respondent answers whether they have had such a life event (no = 0, yes = 1), and whether the respondent thinks it is still affecting him or her (no = 0, yes = 1). The scores are summed, with a higher score indicating more stressful events and a stronger effect.

### Statistical Analysis

All analyses were done using the SAS (V.9.4) statistical analysis system. Infant and maternal demographic characteristics (maternal age, marital status, education level, employment status, infant gender) and clinical characteristics of the mothers and infants (MINI Psychiatric diagnosis, EPDS, RLEQ) were described using frequencies, percentages or means and standard deviations. The occurrence of SP moments was described with frequencies (%) and the duration and number of SP moments were graphically depicted.

Bayley's subscales (cognitive, receptive and expressive language, fine and gross motor, socio-emotional, and adaptive) at 18 months were described using means and standard deviations. The number and percentage of delayed infants at 18 months were provided.

All modelling with the occurrence of SP as outcome were done using univariate log-Binomial models. Least square means for each covariate category were reported. The risk ratios (RRs with 95%CI) were reported as effect measure. All modelling with the number of SP moments were done using linear regression and the regression estimate (slope) and standard errors and *p*-values were reported.

For each subscale of the Bayleys' outcomes, the differential between subscale scores at 18 months were examined relative to the scores at 6 months (motor score at 18 months—motor score at 6 months). The average difference (95%CI) was given as a measure of effect. The average differences were compared between the SP and no SP groups (Chi-Square and *p*-values were provided). All models assumed a significance level of 0.05.

### Ethical Approval

Study approval was obtained from the Health Research Ethics Committee (HREC) of Stellenbosch University (Ref #: S12/04/111A) and the Western Cape Provincial Department of Health. Participation was voluntary, and all mothers provided written informed consent. As the children were under 18 months, assent was provided by the parent. Participation or non-participation in the study had no effect on the standard care offered to mothers and their infants. Participants were screened by the research assistant, when flags were raised, or concerns were noticed they were referred to the follow up psychiatric clinic at Stikland Hospital or referred to the psychologist. All data were anonymized using a study number to code for each mother–infant dyad.

## Results

**Figure 1:**
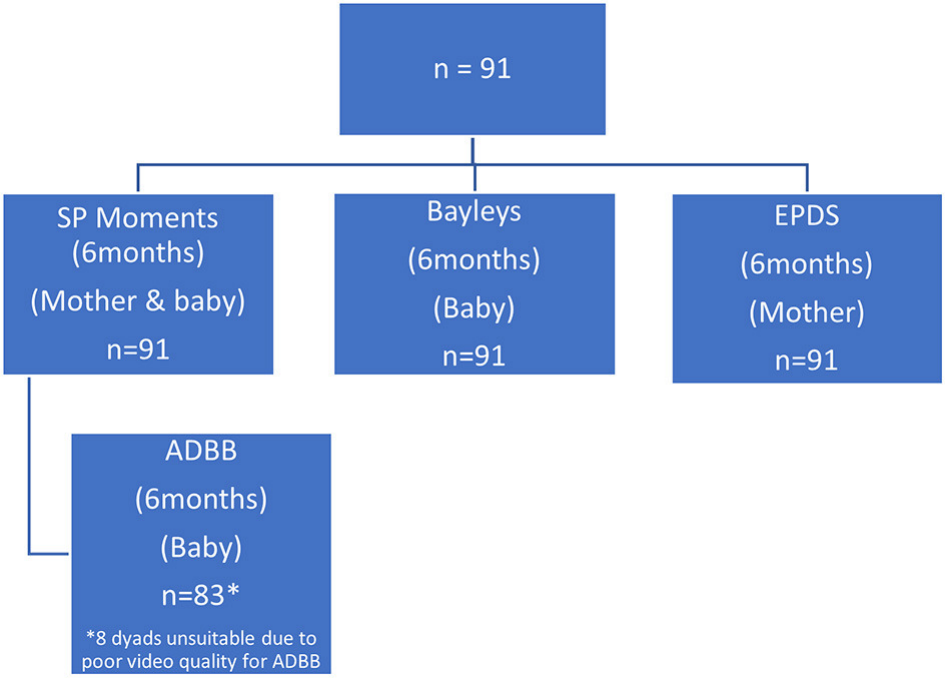
Flow chart of Mother—Baby assessments at 6 months.

Out of 117 study participants, 91 (78%) dyads were eligible for inclusion and could be assessed for dyadic SP (Figure 1). Sixty five percent of mothers were between 17 and 33 years of age, with 35% being older than or equal to 34 years old. The mean age of the mothers was 31 years (SD 5.6). The majority of mothers (59%) in this sample were either divorced or single parents, and 90% of the mothers had completed at least a primary or secondary school level of education. Demographic and clinical characteristics of the dyads (*n* = 91) who participated in this study are summarised in Table 1.

**Table 1:** Demographic^*^ and clinical characteristics of mothers (*n* = 91) of babies at age 6 months.

**Characteristics**	**Variable frequency, *n* (%) or mean (SD)**
Maternal age (years)	Mean 31.1 (SD 5.6)
17–33	59 (65%)
≥34	32 (35%)
**Ethnicity**	
Caucasian/Mixed ancestry	48 (53%)
Black/African	43 (47%)
**Marital status**	
Married	37 (41%)
Divorced/Single	54 (59%)
**Education level**	
Primary/Secondary	82 (90%)
Tertiary	9 (10%)
**Baby gender**	
Male	49 (54%)
Female	42 (46%)

* *Demographic information from mothers were from their first research visit*.

The parent (MIMH) study assessed maternal clinical variables to investigate its impact on infant and relational outcomes. Table 2 presents the maternal psychiatric diagnoses (using the MINI Psychiatric screen) from the first visit, and subsequent 6-month screens of depression (via EPDS) and recent life events (via RLEQ). At the baseline visit, five of the participants did not have a recorded measurement of psychiatric illness. Nine participants had more than one recorded psychiatric diagnosis. Thirty seven percent of the sample did not meet criteria for any psychiatric illnesses, but 41% fulfilled criteria for a major mood disorder and 15% fulfilled criteria for a major psychotic disorder. At 6 months, 73% percent of the mothers scored below the threshold (less than total score of 12) for depression using the EPDS. Using the RLEQ, mothers reported having at least 4 recent life events that had an enduring effect over the last 12 months.

**Table 2:** Maternal clinical variables.

**Clinical parameters**	**Variable frequency, *n* (%) or mean (SD)**
^*^MINI psychiatric diagnoses at 1st visit	
No diagnoses	34 (37%)
Mood disorders	37 (41%)
Psychotic disorders	14 (15%)
Anxiety disorders	11 (12%)
Edinburgh post natal depression score at 6 months (EPDS)	Mean 8.1 (SD 6.7)
Depressed ≥ 12	25 (27%)
Sub threshold <12	66 (73%)
Recent life events questionnaire at 6 months (RLEQ)	Mean 4.5 (SD 4.3)
Number of events	Median = 4

* *5 of the 91 mothers did not have psychiatric illness measurement*.

### Shared Pleasure (SP) Moments

**Figure 2:**
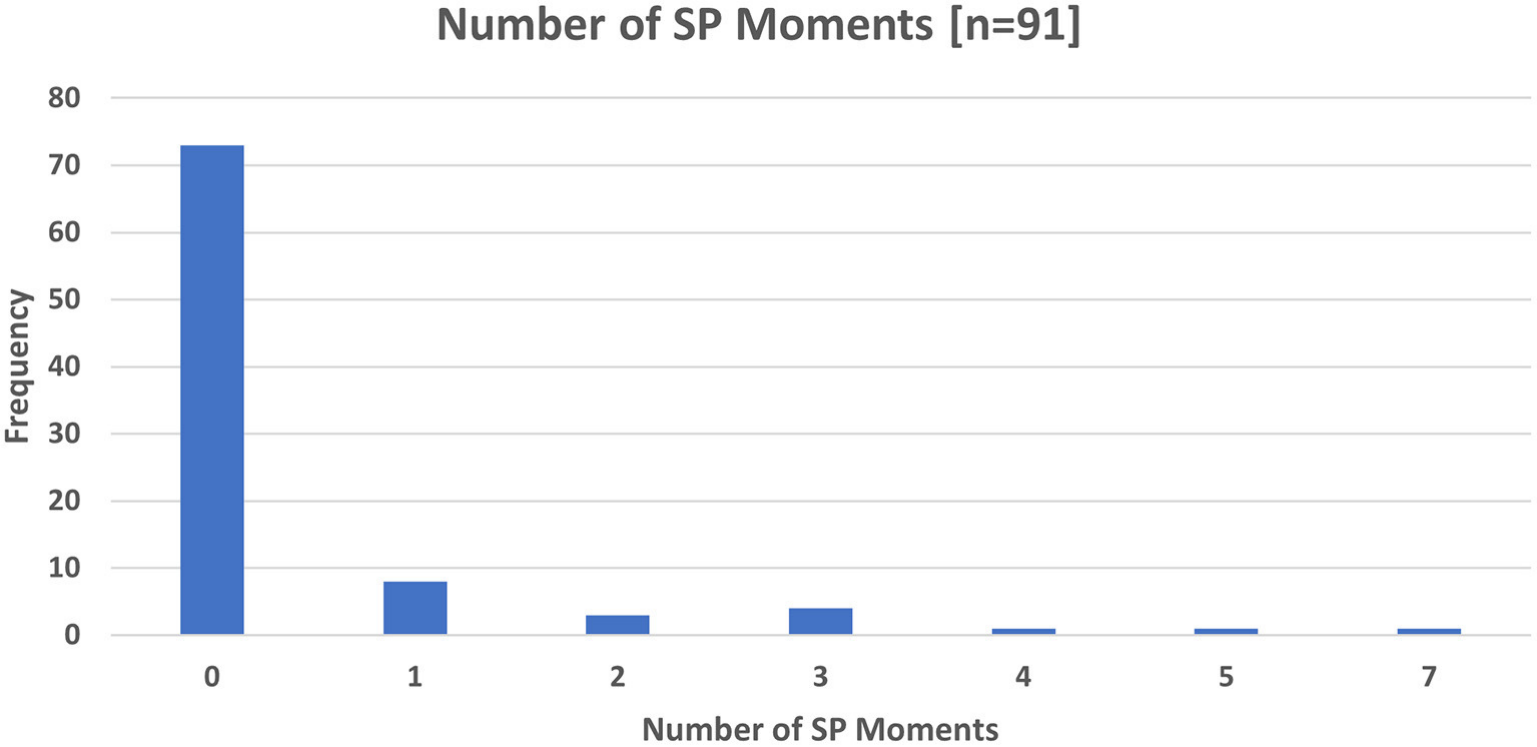
Number of SP Moments at 6 months.

Of the 91 participants, 80% (*n* = 73) did not experience a SP moment. Amongst those who did experience a SP moment (20% *n* = 18), the maximum number of SP moments recorded in a single dyad was 7 (Figure 2).

**Figure 3:**
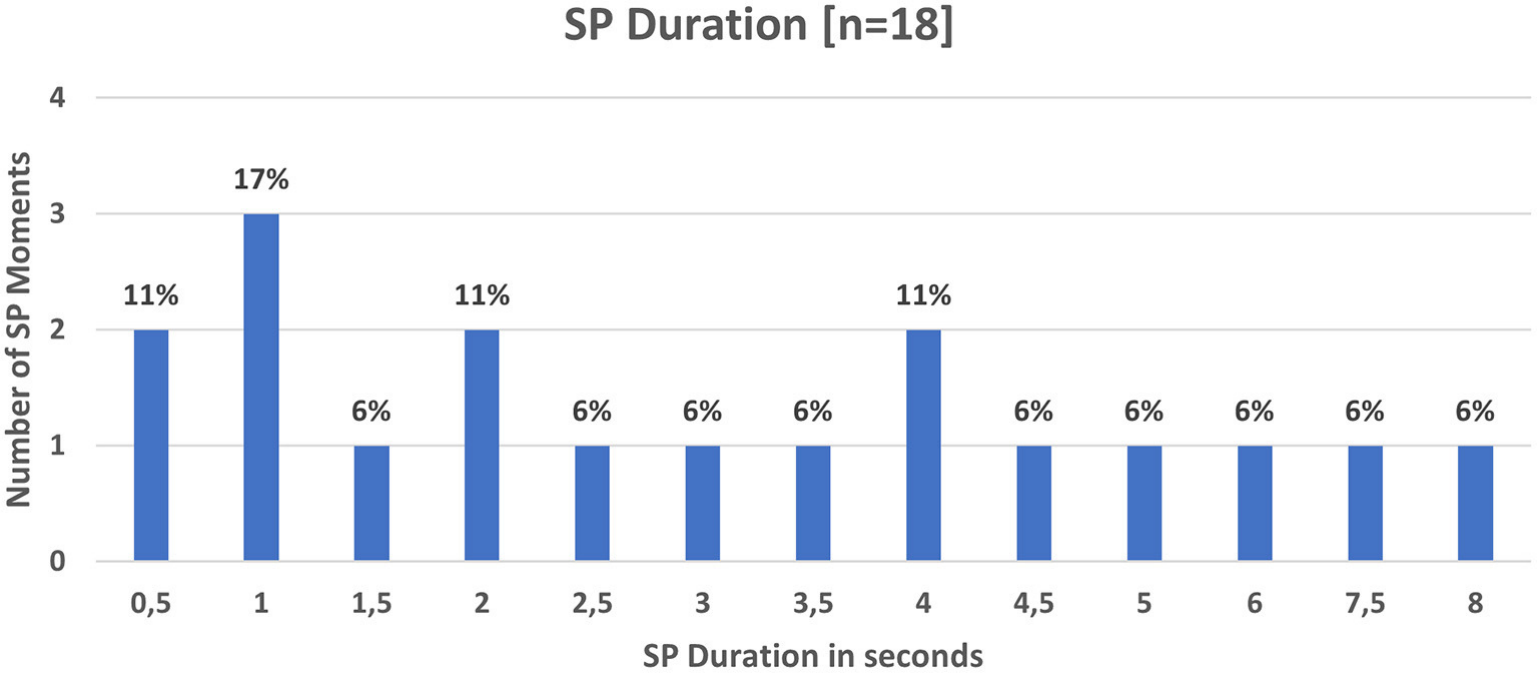
SP moments Frequency and duration (*n* = 18).

A single SP moment lasted at least 0.5 s with the longest duration of an SP moment lasting 8 s (Figure 3). The modal (17%) duration of SP moments was 1 s.

Twenty-two percent (*n* = 13) of mothers younger than 34 years had an SP moment, while 16% (*n* = 5) of older mothers had an SP moment (*p* = 0.46).

There was no significant difference between the age groups (*p* = 0.457). Thirty percent (*n* = 13) of Black African mothers had an SP moment, while only 10% (*n* = 5) of Caucasion/Mixed ancestry mothers experienced an SP moment. Significantly more Black African mothers had SP moments compared to Caucasion/Mixed ancestry mothers (*p* = 0.017).

Of the 34 mothers without a psychiatric diagnosis, 10 (33%) experienced SP moments. The breakdown of SP occurrences in mothers with psychiatric diagnosis are presented in Table 3. None of the 3 psychiatric disorder groups vs. the no psychiatric disorder group were statistically different for the percentage of SP moment occurrences (*p* > 0.05).

**Table 3:** Maternal psychiatric illness and SP.

**Psychiatric diagnosis (MINI) at baseline**	**Number of mothers**	**Number with SP moments (%)**
^*^No diagnosis	34	10 (33.0%)
Mood disorders	37	5 (13.5%)
Psychotic disorders	14	1 (7.1%)
Anxiety disorders	11	4 (36%)

* *Of the 5 mothers with no psychiatric diagnosis, 1 had a SP moment and 4 did not have an SP moment*.

Looking specifically at depression in mothers, 16% of depressed mothers (EPDS score ≥12) experienced an SP moment whereas 21% of mothers who were not depressed experienced an SP moment. There was no significant relationship between having depression and experiencing an SP moment (*p* = 0.571). There was a very weak negative correlation between the EPDS total score for depression and the number of SP moments (Spearman correlation coefficient = −0,186). There was a very weak negative correlation between the number of SP moments and number of events on the RLEQ at 6 months (*r*_s_ = −0.174), as well as a moderate negative correlation with the number of events on the RLEQ at 18 months (*r*_s_ = −0.404).

### Alarm Distress Baby (ADBB) Scale of Infant Withdrawal at 6 Months

From the original sample of 91 participants, only 83 dyads were suitable for assessment of infant withdrawal using the Alarm Distress Baby Scale due to the quality of the early videos in the study, which did not allow for the full assessment of the ADBB categories. Twenty eight percent (*n* = 23) of dyads had an ADBB score in the normal range (score ≤ 5) and 72% (*n* = 60) scored in the withdrawn (score>5) category (Table 4).

**Table 4:** ADBB Scoring (*n* = 83).

**Scoring range**	***N* (%)**
**Normal**	
(≤ 5)	23 (28)
**Withdrawn behaviours**	
> 5 <10	41 (49)
>10	19 (23)

Infant withdrawal (ADBB score > 5) was associated with infant gender. Boys had a significantly higher ADBB score (mean = 9.1; 95%CI: 7.9–10.3) compared to girls (mean = 7; 95%CI: 5.7–8.3) (*p* = 0.025). There was no correlation between the ADBB score and the total score on the EPDS (Pearsons correlation coefficient = 0.09).

### SP and Infant Withdrawal

**Figure 4:**
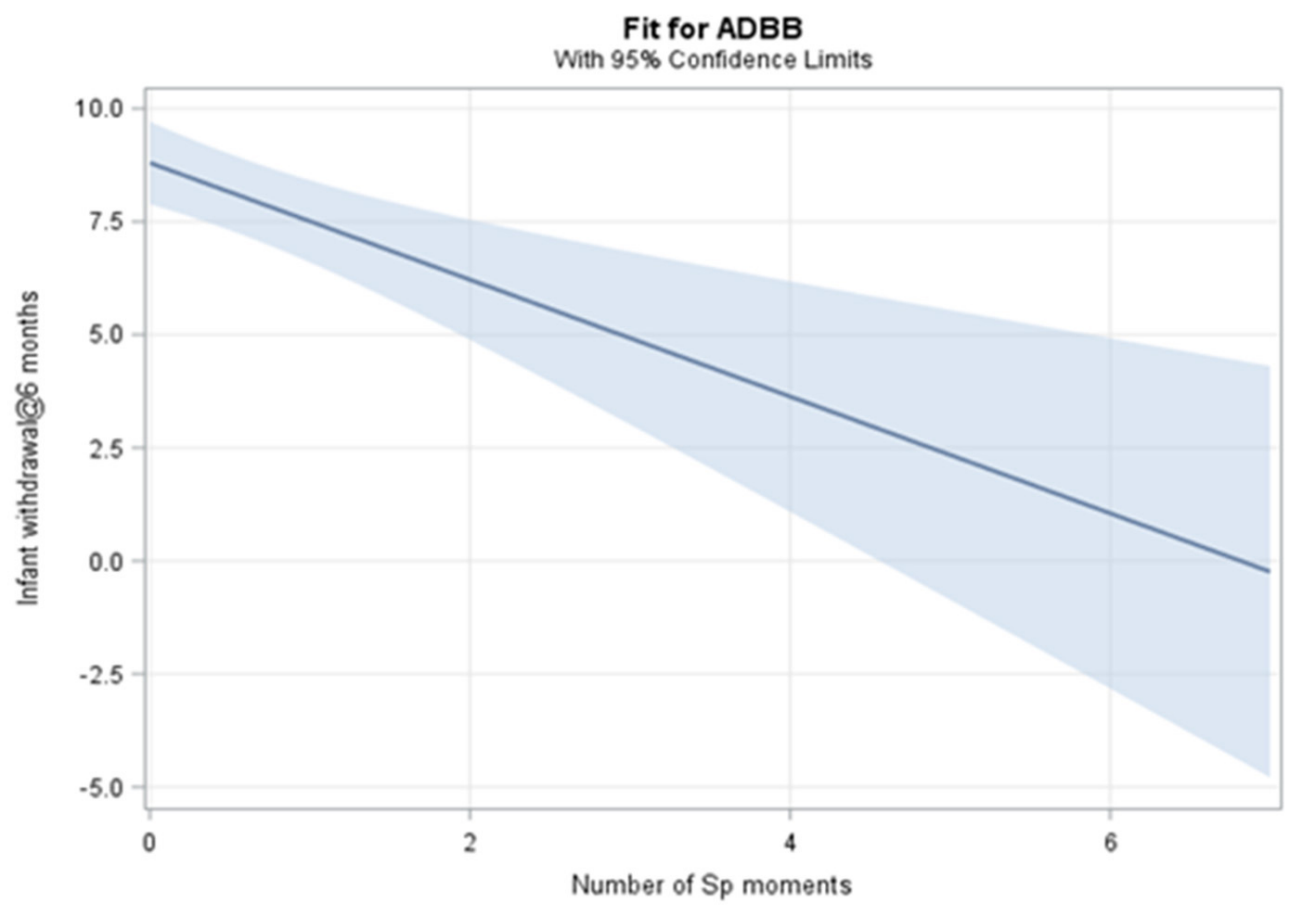
SP moments and association with Infant Withdrawal.

There was a significant linear association (estimate = −1.29; 95%CI: −1.97 to −0.60; *p* = 0.0002) between the number of SP moments and the ADBB score. Figure 4 illustrates how the ADBB score decreases as the number of SP moments increase with the association suggesting that with every additional SP moment the ADBB score decreases by 1.29.

### Infant Development

Infants were assessed using the BSID-III at 6 and 18 months. While 91 infants were assessed at 6 months, only 86 continued to follow up for assessment at 18 months. Composite scores were categorised with a score of <85 indicating a “delay” which was 1 SD from the mean for the South African population (59). At 6 months the majority of infants scored in the normal range for the composite motor (98%), cognitive (96%) and language (98%), adaptive behavioural (92%) and socio-emotional (100%) subscales (Table 5). For those whose scores were available (*n* = 86) for follow up at 18 months, the majority of infants also scored in the normal range for all BSID-III subscales.

**Table 5:** BSID-III Composite Developmental Scores at 6 and 18 months.

**Subscales**	**BSID-III composite score 6 months *n*, (%) (*n =* 91)**	BSID-III composite score 18 months *n*, (%) (*n =* 86)^*^
**Motor**		
No delay (≥85)	89 (98)	81 (94)
Mild/moderate (≥75)	1 (1)	1 (1)
Severe delay (<75)	1 (1)	4 (5)
**Cognitive**		
No delay (≥85)	87 (96)	83 (97)
Mild/moderate (≥75)	3 (3)	3 (3)
Severe delay <75	1 (1)	0
**Language**		
No delay (≥85)	89 (98)	83 (97)
Mild/moderate (≥75)	2 (2)	2 (2)
Severe delay (<75)	0	1 (1)
**Adaptive behavioural**		
No delay (≥85)	84 (92)	74 (86)
Mild/moderate (≥75)	7 (8)	10 (12)
Severe delay (<75)	0	2 (2)
**Socio-emotional**		
No delay (≥85)	91 (100)	86 (100)
Mild/moderate (≥75)	0	0
Severe delay (<75)	0	0

* *missing frequency at 18 months due to no follow up attendance*.

### Shared Pleasure, Depression, and Infant Withdrawal in Relation to Infant Development

Table 6 demonstrates the relationships between the 6-month SP, ADBB and EPDS scores and the BSID-III composite subscales at 18 months.

**Table 6:** SP, EPDS, ADBB 6 months, and BSID-III Composite Scores 18 months.

	**Motor**			**Cognitive**			**Language**			**Adaptive behaviour**			**Socio emotional**		
	**18 months**			**18 months**			**18 months**			**18 months**			**18 months**		
	**Est**.	**95%** **CI**	***p***	**Est**.	**95%** **CI**	***p***	**Est**.	**95%** **CI**	***p***	**Est**.	**95%** **CI**	***p***	**Est**.	**95%** **CI**	***p***
ADBB 6 months	−0.34	−0.81 to 0.14	0.169	−0.38	−0.93 to 0.17	0,176	−0.77	−1.23 to −0.32	^*^0.001	−0.76	−1.31 to −0.21	^*^0.007	−0.58	−1.03 to −0.13	^*^0.012
Number of shared pleasure 6 months	2.37	0.67–4.06	^*^0.006	1.86	−0.02 to 3.74	**0.052**	1.65	−0.004 to 3.30	0.0506	2.12	0.13–4.11	^*^0.037	1,08	−0.54 to 2.68	0,191
EPDS 6 months	0,187	−0.13 to 0.51	0.249	0.20	−0.15 to 0.55	0.258	0,256	−0.03 to 0.57	0.079	0.398	0.04–0.76	^*^0.31	−0.03	−0,33 to 0.26	0.822

* *Significant at a p < 0.05 are indicated in bold*.

The number of shared pleasure moments at 6 months was significantly associated with a higher motor score (est = 2.37; 95%CI: 0.67–4.06; *p* = 0.006), adaptive behaviour score (est = 2.12; 95% CI: 0.13–4.11; *p* = 0.037) and marginally significantly associated with a higher cognitive score (est = 1.86; 95%CI: −0.02 to 3.74; *p* = 0.052) at 18 months.

The ADBB score at 6 months was significantly negatively associated with the adaptive behavioural score at 18 months (est = −0.76; 95%CI: −1.31 to −0.21; *p* = 0.007), the socio-emotional score at 18 months (est = −0.58; 95%CI: −1.03 to −0.13; *p* = 0.012), and also significantly negatively associated with the language score at 18 months on the BSID-III (est = −0.77; 95%CI: −1.23 to −0.32, *p* = 0.001).

There were no significant associations between the EPDS at 6 months and infant motor, cognitive, or language scores at 18 months. A higher EPDS score at 6 months was significantly associated with a higher adaptive behavioural score at 18 months (est = 0.398; 95% CI: 0.04–0.76; *p* = 0.031).

#### Modelling Differential Outcomes

For each subscale of the BSID-III outcomes, the differential between subscale scores at 18 months were examined relative to the scores at 6 months (motor score at 18 months—motor score at 6 months).

The differences were statistically significant (>0) for the motor, cognitive and socio-emotional subscales but not for adaptive behaviour and language subscales (Table 7). For the socio-emotional subscale there was an increase of 5 points from 6 to 18 months, for the cognitive subscale there was an increase of almost 12 points and for the motor subscale there was a decrease of 9 points.

**Table 7:** Observed differences in BSID-III Subscale Outcome (18–6 months).

**Variable**	**N**	**Mean**	**Std Dev**	***p***
Motor difference	86	−9.1	12.96	<0.0001
Cognitive difference	86	11.7	10.50	<0.0001
Adaptive behaviour difference	86	−2.3	11.27	0.062
Language difference	86	−1.3	9.11	0.174
Socio–emotional difference	86	5.0	10.71	<0.0001

For the three subscales for which significant differences were observed, a logistic regression analysis tested for a mean difference between the group with SP moments and the group with no SP moments (Table 8). The observed statistics are summarised in Tables 7, 8:

**Table 8:** Likelihood ratio and Least Squares results for BSID-III and SP outcomes.

**BSID-III Subscale/Type 3 analyses**	**SP presence**	**Estimate (SE)**	**Likelihood ratio chi square**	***p*-value**
Motor difference	No SP	−10.6 (1.5)	4.89	0.027
	With SP	−3.0 (3.0)		
Cognitive difference	No SP	10.4 (1.2)	6.33	0.0118
	With SP	17.4 (2.4)		
Adaptive behaviour difference	No SP	5.1 (1.3)	0.02	0.8988
	With SP	4.7 (2.6)		

#### Differences in Motor Scores

A significant difference was observed in motor scores at 18 months vs. at 6 months with the mean difference being −9.1 (SD 10.50), indicating that on average the 18-month scores were worse than the 6-month scores. The logistic regression results show that the average decrease in motor scores is larger for the group without SP (10.6) compared to the group with SP (3). The likelihood ratio demonstrates that the absence of SP moments is 4.89 times more likely to produce a negative difference (i.e., a worse motor outcome) at 18 vs. 6 months.

Similarly, Table 8 shows that while the average motor score is worse at 18 months regardless of the presence of SP moments, the average motor scores differentials are significantly worse (*p* = 0.027) at 18 months if SP moments are absent (−10.6) versus if they are present (−3.0).

#### Differences in Cognitive Scores

A significant difference was observed in cognitive scores at 18 months vs. at 6 months with the mean difference being 11.7 (SD 12.97), indicating that on average the 18-month scores were better than the 6-month scores. The likelihood ratio demonstrates that the presence of SP moments is 6.33 times more likely to produce a positive difference at 18 vs. 6 months (i.e. a better cognitive outcome).

Similarly, Table 8 shows that while the average cognitive score is better at 18 months regardless of the presence of SP moments, the average cognitive scores differentials are significantly better (*p* = 0.0118) at 18 months if SP moments are present (17.4) vs. if they are absent (10.4).

### Differences in Socio-Emotional Scores

A significant difference was observed in socio-emotional scores at 18 months vs. at 6 months with the mean difference being 5.0 (SD 10.71), indicating that on average the 18-month scores were better than the 6-month scores. The likelihood ratio demonstrates no significant difference in socio-emotional outcome linked to the presence or absence of SP moments. Similarly, Table 8 shows no significant difference (*p* = 0.898) in the average socio-emotional score differentials at 18 months if SP moments are absent (5.1) vs. if they are present (4.7).

## Discussion

The present study assessed SP moments in early interactions between 91 mothers and their 6-month-old infants and its associations with infant withdrawal behaviours. The contribution of SP to later developmental outcomes at 18 months was also examined in a subset of 86 infants.

### Shared Pleasure

The low occurrence of SP (20%) at 6 months was consistent in that the same low occurrence of SP was observed when infants were 2 months old (31). This is much lower when compared to studies in Europe which reported higher rates (above 60%) of SP, although these studies were conducted in community non-clinical samples (29, 60). We speculate that infants in LMIC's who are disproportionately vulnerable to socioeconomic and environmental adversities (8) are less likely to experience positive engagement with their mothers who themselves are at risk for multiple stressors. While there is no one single style of parenting that can characterise mothers as a group, life circumstances and experiences may contribute to shaping the nature and quality of the engagement (61). Maternal age did not influence the experience of SP between mothers and their infants (*p* = 0.457). Black African mothers experienced significantly more SP moments (*p* = 0.017) than their Mixed ancestry/Caucasian counterparts. This may speak to many postulated cultural factors such as a strong intergenerational transmission of positive parenting styles (62) or the social contexts in which Black mothers shape their perspectives around positive sensitive mothering (63). However, given the small sample and the diversity of community cultural constructs in South Africa, it is difficult to assume that all mothers of a particular community experience or practise similar caregiving. In addition, infants are often co-parented in this setting by more than one adult caregiver which may influence the infant's socialisation experiences. The co-ordinated co-parenting dynamic in families may fundamentally influence the young infants' early social and acculturation experience as described by McHale et al. (64).

At baseline, 41% of mothers in the parent study had been diagnosed with a mood disorder. At the 6-month baby visit, 73% of mothers did not report any symptoms of depression. The occurrence of an SP moment was not significantly associated with depressive symptoms measured by the EPDS (*p* = 0.571). This is similar to other studies of SP in Europe which did not find an association between depressive symptoms measured by the EPDS and SP (30). While there are studies that show maternal depression specifically influencing the parents' capacity to provide adequate stimulation or to respond adequately to the infant (65, 66), other factors such as infant temperament or maternal maturity can also influence the interaction. The infant itself plays an important role in this relationship (67). For example it is possible that a highly empathetic infant may persistently attempt to engage with a depressed mother by smiling and striving for eye contact. It has in fact been suggested that the infant of a depressed mother might become exceedingly sensitive to the mother's emotional state in order to read her better and to better regulate the interaction (68). The family interactional dynamics and co-ordinated parenting efforts may also influence the infant's social responses to the mother's depression. This may include learned social interactions within the family alliance such as participation and affect sharing (69).

While depression may not have been associated with lowered levels of the positive reciprocal interactions, economic stress, and disadvantage could also be considered to exacerbate the residual effects of depression and demands on parenting by mothers feeling more physically and emotionally strained (70). In this study, mothers reported on average 4.5 (SD = 4.3) stressful life events having an enduring effect during the past 12 months. Mothers who have previously been depressed may even find engaging in positive parenting interactions more challenging, with exposures to psychosocial stressors moderating this effect (70). We noted a moderate negative correlation between having an SP moment at 6 months and the number of life events at 18 months (*r*_s_ = −0.404), supporting the suggestion that stressful life events themselves are associated with impaired parent infant interactions (71). This was somewhat similar to a Finnish study which found a negative correlation (−0.23) between parental stress and the presence of SP (30), although a later study by the same researchers when using the LEQ, found no significant relationship between number of life events and the presence of SP (29).

### Infant Withdrawal

Given the low occurrence of positive SP interactions as a measure of synchrony and engagement between the dyad, it was expected that there would be a high occurrence of withdrawn infant behaviours in this cohort. Seventy two percent (*n* = 60) of those interactions that could be coded with the ADBB, scored in the risk of concern for infant withdrawal (i.e., an ADBB score >5). This is much higher than rates of infant withdrawal reported in high income countries (20) but in keeping with the higher reported rates in South African infants with HIV (31%) (35), infants with Foetal Alcohol Syndrome (27%) (51), and infants from a high risk community (46.7%) (49). More concerning, 23% of these infants scored in the very high risk for severe infant withdrawal (ADBB>10). This is similar to other studies in high or at risk infant populations who were exposed to environmental adversities including socioeconomic stressors and maternal mental health risks (32, 72) and who responded less interactively in engagements with their mothers. Infant withdrawal was associated with a gender difference, in that male infants had a significantly higher ADBB score (mea*n* = 9.1) compared to female infants. This mimics similar findings by (29) and may be explained by the fact that male infants (due to sex hormones, endocrine alterations and social experiences) may experience a later differential maturation of the brain (73, 74). This may confer more vulnerability to the male infant that is exposed to stressors in the social environment and during the regulation of socio-emotional engagement the mother (68, 74). Similar to other South African studies (35, 49), there was no correlation between maternal depression and infant withdrawal in our study. However, there was a significant linear association (*p* = 0.0002) between the number of SP moments and infant withdrawal, where the ADBB score decreased by 1.29 points, with each additional SP moment. This makes sense in that the more interactive and synchronised the dyadic engagement is, the less likely it is for an infant to show features of withdrawal. This echoes findings of Puura et al. (29) who reported a lack of infant withdrawal associated with the presence SP in female infants in their study.

### Infant Development

Using the BSID-III, the majority of infants in this study fell into the normal developmental range at 6 months, and this persisted for most at the 18-month follow up assessments. It is well-documented that positive features within the early mother infant relationship should be supported due to their association with better infant developmental outcomes (75, 76). In this sample, the number of SP moments experienced by infants at 6 months was significantly associated with a higher motor score (*p* = 0.006), adaptive behaviour score (*p* = 0.037) and cognitive score (0.052) at their 18-month developmental assessment. Mother-infant relationship synchrony is considered an important determinant of infant outcomes (19). The contribution of SP in this sample to later developmental outcomes support the theory that early positive emotional engagement expressed during joyful circumstances may account for better later infant development (77).

Similar to findings in other populations of withdrawn infants (43, 44), our study found significant negative associations between ADBB scores at 6 months and later language (*p* = 0.001), adaptive behavioural (*p* = 0.007) and socio-emotional (*p* = 0.012) composite scores at 18 months. Excessive social withdrawal can disrupt the ability of the infant to interact adequately and reciprocally with their caregivers, which is necessary for the normal development of effective social communication and emotional responsiveness by the infant (34, 78). Early infant withdrawal may serve as a risk indicator for later socio-emotional disorders (79), and as our study shows, this can be seen in later impaired adaptive behavioural and socio emotional developmental competencies.

While there were no significant associations between EPDS and motor, cognitive or language scales of development, an interesting result was the significant association between a high maternal EPDS depression score at 6months and higher infant adaptive behavioural composite scores at 18 months (*p* = 0.031). The lack of associations between early postpartum depression and objective assessments of motor, cognitive, and language developmental scales has been commonly described (80, 81). The adaptive behavioural domain in the BSID III however is based on information supplied by the primary caregiver rather than an objective assessment of the competency of the infant. The behaviour and perceptions of depressed mothers is heterogenous, however Weinberg and Tronick (68) reported that depressed mothers' self-evaluations are not always concordant with their behaviours and interactions with infants, and that their perceptions may in fact not always reflect how their infants are actually performing. This could possibly explain the association here, and that while mothers at risk of depression may have negative perceptions of their own parenting capabilities, they may over-estimate their infants' individual competencies as a way of compensating for their own perceived negative parenting.

### Modelling Outcomes

Studies in LMICs inconsistently show associations between maternal mental health disorders and developmental outcomes (81, 82). As significant differences between the 18 -month and 6-month scores for motor, cognitive and socio-emotional scores were noted, we were interested in whether the presence of an SP moment influenced these differences between the scores.

For motor scores, the mean difference between scores at the 2 time points was −9.1 (SD 10.50), with a larger decrease in motor scores noted for the group that did not have an SP moment. In LMICs the accumulation of risk exposures including various environmental stressors across time is likely to affect child development and result in poorer outcomes (82). In our study however, logistic regression modelling suggests that experiencing an SP moment when infants were 6 months appears to confer some protection later in motor developmental outcomes, with a worse motor outcome being more likely in infants who did not experience an early SP moment (*p* = 0.027). The potential for better infant motor developmental outcomes being influenced by positive maternal interactions has been described in other settings (83, 84). Specifically, supporting the influence of early experiences of the high maternal engagement, findings in a longitudinal birth cohort in the UK showed that children whose mothers engaged more with them from early infancy (6 months) had higher gross and fine motor skills 12 months later, particularly significantly for mothers who had lower socio-economic backgrounds (85). Similarly, while our study sample size is much smaller, our results suggest that the potential protective influence of SP (at 6 months) may manifest itself later in moderating developmental outcomes.

Assessing cognitive outcomes, overall improvements were noted across the sample, but modelling showed stronger associations for the presence of SP moments. This included a significantly larger improvement (*p* = 0.018) in the average cognitive score differentials at 18 months if SP moments were present (17.4) vs. if they were absent (10.4). This difference was 6.33 times more likely if there was an SP moment at 6 months. Children's cognitive development is influenced by several factors, including psychosocial exposures like parent-child interactions, cognitive stimulation and shared nurturing learning opportunities (86). The WHO's Nurturing Care Framework highlights the presence of bidirectional communication and enjoyable stimulating care as core to the provision of responsive care within a healthy mother–child relationship. Specifically the positive caregiver-child interaction helps to develop the social and emotional development of the infant. Shared pleasure requires a synchronous and joyful engagement which enhances the shared experience and thereby creates an opportunity for learning which may ultimately support infant cognitive development. As other studies have proposed, a significant positive quality of the mother infant relationship and parenting capacity has potential to contribute to favourable child development attachment (6, 87) and SP in this case, may likely be one of those protective contributors.

For socio-emotional scores, while there were improvements noted overall at 18 months, SP was not shown to significantly impact on the difference in scores from 6 months to 18 months.

## Conclusion

Given the disproportionately high rates of maternal perinatal illness and environmental adversities in LMICs, it is important to explore possible opportunities that may contribute to improving outcomes in infants. This study explored associations between maternal SP interactions, infant withdrawal, and infant developmental outcomes. While the occurrence of SP in this at-risk sample was low and the rates of infant withdrawal were high, there were promising results suggesting that early positive shared pleasure interactions may contribute to improvements in subsequent developmental outcomes. Further more, the SP paradigm could serve as an observation based screening marker for health care workers in the primary care setting where high risk dyads are followed up.

## Limitations

While SP, EPDS, and ADBB were associated with subscales of the BSD-III, due to the small sample size, we could not assess these as intervening variables. This will be explored in the larger sample population of this study. A further limitation include the influence of the parenting roles on the infants' developmental milestones which were not assessed. Additional parameters such as a measure of infant temperament and genetic profiles that could influence infant withdrawal and SP were beyond the scope of this study. The videos available at 18 months to assess infant outcomes were only of the infant alone during the developmental assessment and not of the mother with the infant. Hence dyadic assessments were not possible at 18 months. Additionally, the SP is most sensitive in younger infants, in that older infants (usually older than 7 months) who are able to crawl away or independently explore, are thought to have fewer/less frequent opportunities for spontaneous synchronised direct gaze contact (29). The SP was therefore not assessed at 18 months.

## Data Availability Statement

The raw data supporting the conclusions of this article will be made available by the authors, without undue reservation.

## Ethics Statement

The studies involving human participants were reviewed and approved by Human Subjects Research Ethics Committee, STellenbosch University, FMHS. Written informed consent to participate in this study was provided by the participants' legal guardian/next of kin.

## Author Contributions

AL, KP, DN, and JL contributed to conception and design of the study. EJ performed the statistical analysis. MB performed assessments and data collection. AL wrote the first draught of the manuscript. All authors contributed to manuscript revision, read, and approved the submitted version.

## Conflict of Interest

The authors declare that the research was conducted in the absence of any commercial or financial relationships that could be construed as a potential conflict of interest. The reviewer MF declared a shared committee with one of the authors KP at time of review.
